# Prophylaxis Failure and Successful Management of Delayed-Onset Malaria with Renal Complications: A Case Report with Oral Artemether-Lumefantrine Treatment

**DOI:** 10.3390/reports6040053

**Published:** 2023-11-08

**Authors:** Ilir Tolaj, Gramoz Bunjaku, Murat Mehmeti, Yllka Begolli

**Affiliations:** 1 Department of Infectious Diseases, Medical Faculty, University of Pristina, 10000 Pristina, Kosovo; 2 Department of Infectious Diseases, University Clinical Center, 10000 Pristina, Kosovo; murat_meh@hotmail.com; 3 Department of Clinical Microbiology, University Clinical Center, 10000 Pristina, Kosovo; yllkabegolli@gmail.com

**Keywords:** severe malaria, plasmodium falciparum, chemoprophylaxis failure, oral artemether-lumefantrine

## Abstract

This case report presents a critical clinical scenario involving a 55-year-old patient who developed severe *Plasmodium falciparum* malaria with renal complications despite receiving doxycycline prophylaxis while traveling in a malaria-endemic region. The case emphasizes the potential failure of doxycycline prophylaxis and highlights the importance of considering malaria in patients with a history of travel to endemic areas, even if they have adhered to prophylactic treatment. The patient’s clinical presentation included fever, extreme fatigue, and loss of consciousness, leading to hospitalization. Laboratory findings revealed severe anemia, elevated liver enzymes, and impaired renal function, consistent with the criteria for severe malaria. The diagnosis was confirmed by the presence of *Plasmodium falciparum* parasites on thin blood smears. Due to limited access to parenteral antimalarial medications in Kosovo, the patient received oral artemether-lumefantrine, resulting in clinical improvement. Supportive care and dialysis played a vital role in the patient’s recovery. This case report underscores the need for increased awareness of prophylaxis failure, the challenges of managing severe malaria in non-endemic countries, and the importance of timely and appropriate interventions to improve outcomes in severe malaria cases, particularly those with renal involvement.

## 1. Introduction

Malaria, caused by Plasmodium parasites and transmitted through mosquito bites, remains a significant global health concern, particularly in regions with high endemicity. In 2021, there were an estimated 247 million malaria cases worldwide, marking an increase from the 2020 count of 245 million, primarily observed in countries within the WHO African Region. In 2015, the Global Technical Strategy for Malaria 2016–2030 (GTS) set a baseline of 230 million cases, aiming for a reduction of at least 75% in case incidence and mortality rate by 2025, and 90% by 2030 from the 2015 baseline. However, the GTS 2020 milestones for morbidity and mortality were not met globally, and current trends suggest that the 2025 targets are also at risk. The observed malaria case incidence of 59 cases per 1000 population at risk in 2021 deviates significantly from the expected 31 cases per 1000, indicating a 48% deviation from the set goal [1].

The signs and symptoms of malaria are non-specific, primarily characterized by fever or a history of fever. However, relying solely on clinical features for diagnosis lacks specificity and often leads to overtreatment. Travelers acquiring malaria, particularly those from non-endemic areas, face distinct challenges. They are typically non-immune individuals residing in urban settings of endemic countries or visitors from non-endemic regions. When these travelers return to non-endemic countries and present with malaria, case fatality rates tend to be higher due to delayed diagnosis, unfamiliarity with malaria among healthcare professionals, and limited availability of effective antimalarial drugs. This underscores the critical need for awareness and preparedness in non-endemic areas [2].

In all settings, suspected malaria should be confirmed with a parasitological test, and the result should be available within 2 h of the patient presenting. In settings where parasitological diagnosis is not possible, a decision to provide antimalarial treatment must be based on the probability that the illness is malaria. The two methods used routinely for parasitological diagnosis of malaria are light microscopy and immunochromatographic rapid diagnostic tests (RDTs) [2]. 

In falciparum malaria, the risk for progression to severe malaria with vital organ dysfunction increases at higher parasite densities. In low-transmission settings, mortality begins to increase when the parasite density exceeds 100,000/μL (~2% parasitemia). Patients with a parasitemia of 4–10% and no signs of severity also require close monitoring, and, if feasible, admission to hospital. They have high rates of treatment failure. Non-immune people such as travelers and individuals in low-transmission settings with parasitemia > 2% are at increased risk and also require close attention. Parasitemia > 10% is considered to indicate severe malaria in all settings [2]. 

WHO gives specific recommendations for the treatment of different clinical forms of malaria. A patient who presents with symptoms of malaria and a positive parasitological test (microscopy or rapid diagnostic test - RDT) but with no features of severe malaria is defined as having uncomplicated malaria. The clinical objectives of treating uncomplicated malaria are to cure the infection as rapidly as possible and to prevent progression to severe disease. “Cure” is defined as the elimination of all parasites from the body. Artemisinin-based combination therapy (ACT) is strongly recommended, with high certainty evidence. Children and adults with uncomplicated *P. falciparum* malaria should be treated with one of the following ACTs: artemether-lumefantrine (AL), artesunate-amodiaquine (AS + AQ), artesunate-mefloquine (ASMQ), dihydroartemisinin-piperaquine (DHAP), artesunate + sulfadoxine-pyrimethamine (AS + SP) and artesunate-pyronaridine (ASPY); AS + SP and ASPY are not recommended for use in the first trimester of pregnancy. A 3-day course of the artemisinin component of ACTs covers two asexual cycles, ensuring that only a small fraction of parasites remain for clearance by the partner drug, thus reducing the potential development of resistance to the partner drug. Recommendations for special situations and special risk groups are also given [2]. Severe malaria is defined as one or more of the WHO-listed clinical criteria, occurring in the absence of an identified alternative cause and in the presence of *P. falciparum* asexual parasitemia. Severe malaria is treated by promptly giving full doses of effective parenteral (or rectal) antimalarial treatment. This should be followed by a full dose of effective ACT orally. Two classes of medicine are available for the parenteral treatment of severe malaria: artemisinin derivatives (artesunate or artemether) and cinchona alkaloids (quinine and quinidine). Parenteral artesunate is the treatment of choice for all severe malaria. The current recommendation of experts is to give parenteral antimalarial drugs for the treatment of severe malaria for a minimum of 24 h once started (irrespective of the patient’s ability to tolerate oral medication earlier) or until the patient can tolerate oral medication, before giving the oral follow-up treatment. After initial parenteral treatment, once the patient can tolerate oral therapy, it is essential to continue and complete treatment with an effective oral antimalarial drug by giving a full course of effective ACT (artesunate + amodiaquine, artemether + lumefantrine or dihydroartemisinin + piperaquine) [2]. 

The distribution of drug-resistant *P. falciparum* nowadays remains variable across the globe, reflecting in part patterns in drug deployment and transmission intensity. Resistant to two of the ACTs, DHA-PPQ and AS-MQ, are well documented in Southeast Asia. Reports of ACT treatment failures in malaria-naïve travelers who contracted *P. falciparum* malaria in sub-Saharan Africa echo the first report of chloroquine (CQ)-resistant malaria, though none contain definitive confirmation of resistance and at least two of the reports included failures attributable to subtherapeutic dosing rather than drug resistance. Evidence of genotypic and phenotypic correlates of resistance to one or more ACT components is beginning to emerge in sub-Saharan Africa, South of Asia, and South America, including a recent report from Rwanda where *P. falciparum kelch13 (pfk13)* R561H, P574L, and C469Y alleles, previously linked to a clearance phenotype, were detected. Resistance to the antifolates remains widespread, while reversion of CQ-resistant parasite populations to the CQ-susceptible wild type followed in the wake of withdrawing CQ from national formularies in eastern and central-southern Africa [3].

In regions with high CQ or multidrug resistance, doxycycline at 100 mg, administered one day prior to travel, daily during travel, and continued for four weeks post-return, is a recommended chemoprophylactic regimen [2]. Studies on compliance with chemoprophylactic regimens suggest that regular use of appropriate chemoprophylaxis is effective when taken consistently [4,5]. Pre-travel advice for drug prophylaxis should highlight the fact that correctly taken antimalarials provide efficacy against *P. falciparum* that comes close to 100% [6]. However, prophylactic failures are attributed to inadequate dosing, patient non-compliance, and emerging resistance [7,8,9,10,11].

WHO recommends two vaccines against malaria; RTS,S/AS0, which received a WHO recommendation in 2021, and R21/MatrixM vaccine, which received approval this year. Both vaccines are shown to be safe and effective in preventing malaria in children and, when implemented broadly, are expected to have a high public health impact. Both vaccines and future ones should be provided as part of a comprehensive malaria control strategy [2,12]. 

Mortality due to complications of severe malaria depends on multiple factors, such as medical circumstances surrounding the patient, the treatment received, and the course of the infection itself. Patient frailty due to factors not directly associated with malaria, such as age, co-infection with viruses or bacteria, or weak immune system, can lead to death, regardless of the infecting parasite. Severe malaria usually has a mortality rate of more than 5%, which is high compared to uncomplicated malaria cases, where the mortality rate is as low as 0.1% [13]. A cohort study of 400 critically ill adults in France, showed that the intensive care unit mortality rate was 10.5%, while older age, low Glasgow Coma Scale and higher parasitemia were independently associated with hospital deaths [14]. The progression from uncomplicated malaria to severe malaria is linked to the timely initiation of the treatment. If initiation of treatment is delayed or not completed soon enough and the parasite burden progresses, uncomplicated malaria develops into severe malaria [15].

Impaired renal function is an important manifestation of severity in younger children, but acute kidney injury (AKI) requiring renal replacement therapies is almost confined to older children and adults [16].

Acute kidney injury (AKI) is a frequent complication of severe malaria, occurring in approximately 40% of severe *P. falciparum* cases in endemic areas, with a mortality rate of around 75%. The pathogenesis involves mechanisms such as acute tubular necrosis, infection-triggered proinflammatory reactions, and metabolic disturbances. Renal insufficiency typically manifests 3–7 days after fever onset, with serum creatinine improvement taking an average of 17 ± 6 days. Electrolyte imbalances, including acidosis, hyponatremia, and hyperkalemia, are common, and primarily attributed to tissue hypoxia, initial internal dilution, and hemolysis. Additionally, anemia and thrombocytopenia arise from the malarial infection. Some patients develop proteinuria due to glomerulonephritis. *P. falciparum* is the primary causative agent, contributing significantly to AKI cases [17,18,19,20].

Advanced age, referral from another hospital, hyperbilirubinemia, inotropic drug requirement, hospital-acquired secondary infection, hyperkalemia, jaundice, altered consciousness level, leukocytosis, oligo-anuria, and *P. falciparum* infection are identified risk factors for AKI in malaria. Oligo-anuria is a common clinical presentation (46–76% of cases), accompanied by severe metabolic acidosis and hypercatabolic state. Notably, hyponatremia and hyperkalemia are frequent electrolyte abnormalities in malaria-associated AKI, occurring in 30–50% of cases [18].

The primary goal in treating severe malaria is preventing mortality, necessitating prompt administration of effective parenteral antimalarial treatment followed by a full course of oral artemisin-based combination therapy (ACT). For severe cases, parenteral (IV or IM) artesunate is recommended for at least 24 h, switching to oral treatment once tolerated. If artesunate is unavailable, artemether is the preferred alternative over quinine [2]. Supportive care, fluid replacement, renal replacement therapy, and avoidance of nephrotoxic drugs are crucial components in managing malarial acute renal failure (ARF) [21].

This case report highlights the challenges associated with diagnosing and treating severe *P. falciparum* malaria with renal involvement, especially in non-endemic and developing countries, like Kosovo. The delayed-onset symptoms despite doxycycline prophylaxis underscore the need for continued vigilance and effective management strategies, particularly in travelers from non-endemic regions. Implementing timely and appropriate interventions remains critical in improving outcomes for patients with severe malaria and renal complications.

## 2. Detailed Case Description

A 55-year-old patient, without a previous medical history, was admitted to the hospital due to a cough, fever (39 °C), extreme fatigue, and sudden loss of consciousness. Although the patient resides in Pristina, he is a businessman who visited Guinea for a month and a half and returned some ten days ago. The patient was prescribed prophylactic doxycycline 100 mg/day (brand name Dovicin) by the family doctor, which, according to the patient, was taken regularly, starting two days before the trip and continuing throughout his 1.5-month stay in Guinea, and continuing additional 4 days after returning home in Kosovo. During his stay in Guinea, he used bed nets provided by the hotel services, and occasionally mosquito repellants. He did not take any medications that could interact with doxycycline. While still on prophylactic treatment, on day 4 after his return home, he started not feeling well, experiencing a cough and high fever. On day 5 after his return, after an examination by a family physician, the patient was diagnosed with pneumonia, considered to be atypical. On the same day, the doxycycline prophylactic treatment was stopped, and the treatment with azithromycin 100 mg qd started. The condition of the patient deteriorated with irregular high fever, extreme fatigue and sudden loss of consciousness, forcing the patient to be hospitalized, on the 8th day after his return home, and 4 days after he stopped the prophylactic treatment. 

On admission, the patient was alert, Glasgow coma scale of 15, blood pressure was 100/60 mmHg, pulse rate was 98/min, respiratory rate was 20/min, febrile with body temperature 39 °C, and oxygen saturation at 92% on room air. Physical examination revealed a normal lung and heart condition, slightly enlarged liver and spleen, no joint swelling, no skin rash, no enlarged lymph nodes, with signs of moderate dehydration and moderate jaundice of sclera. He noticed less urination in the last two days, but he related this to the high temperature and sweating he had.

The patient’s blood test results on admission to the hospital showed a lower limit of the red blood cell (RBC) count with a value of 3.93 × 10^12^/L (3.80–5.80 × 10^12^/L), which during the first week of treatment decreased to the level of 2.4 × 10^12^/L; hemoglobin (Hgb) level of 9.1 g/dL (11.00–16.5 g/dL), which further decreased to the levels of 6.5 g/dL; platelet levels (PL) of 86 × 10^9^/L (150–390 × 10^9^/L), which further decreased to 77 × 10^9^/L; and white blood cells (WBC) of 9.2 × 10^9^/L (3.5–10.0 × 10^9^/L, which later increased to 17.5 × 10^9^/L. The patient’s C-reactive protein (CRP) was 113.1 mg/dL, later increased to 212 mg/dL; erythrocyte sedimentation rate (ESR) was 20 mm/h, liver enzymes were elevated with aspartate aminotransferase (AST) 90 U/L (8–48 U/L), later increased to 183 UI/L, and alanine transaminase (ALT) 82 U/L, (7–55 U/L) increased to 165 U/L, total bilirubin was 99.9 µmol/L (1.71–20.5 µmol/L and direct bilirubin was 77.3 µmol/L (<5.1 µmol/L), both elevated, later gradually returned to normal; glycemia 3.88 mmol/L (3.9–5.6 mmol/L), which later returned to normal levels; urea (19.45 mmol/L, and later 39.15 mmol/L) and creatinine (223.7 µmol/L, and later 557.8 µmol/L); total proteins/albumins 5.6/3.4 g/dL (6–8/3–5 g/dL), later 4.6/2.5 g/dL; LDH 1075 U/L (normal range 140–280 U/L), later increased to 1897 U/L, and then decreased to 630 U/L; D-dimer 6650 ng/mL (normal <500 ng/mL), decreased to 933 ng/dL; PT 88 s (normal 11–13.5 s); pH 7.37, later 7.41; PaCO_2_ 28–44 mmHg (normal range 35–45 mmHg); PaO_2_ 35–135 mmHg (normal 75–100 mmHg); sodium 133–157 mEq/L (135–145 mEg/L); potassium 3.5–4.1 mmol/L (normal 3.6–5.3 mmol/L); calcium 0.95–1.19 mmol/L (normal 2.13–2.55 mmol/L) (Table 1). 

**Figure 1:**
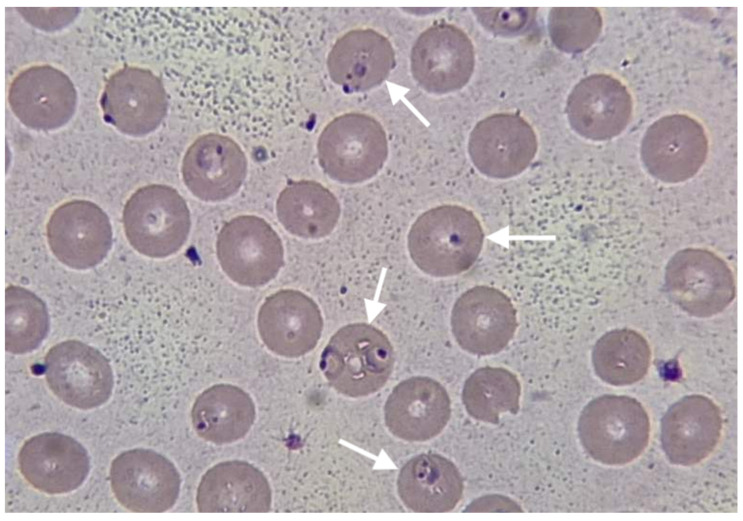
Thin blood smear revealing growing rings and trophozoites of *Plasmodium falciparum* (arrows).

Chest X-ray revealed non-homogeneous opacities in the right lung; abdominal USG revealed hepato-splenomegaly, increased size of the kidney and loss of kidney corticomedullary differentiation. Blood cultures were negative, as well as a rapid immunochromatographic test for IgM/IgG antibodies for Leishmania and Leptospira. The rapid immunochromatographic test for malaria (The Right Sign Malaria Pf test, Biotest, Hangzhou Biotest Biotech Co, China) was positive. Thin blood smear examination revealed growing rings and trophozoites of *Plasmodium falciparum*, (Figure 1). Parasite density was quantified, revealing very high parasitemia at the level of 20%.

The treatment with artemether 80 mg/lumefantrine 480 mg was initiated on the first day of hospitalization, for 6 consecutive days. This regimen, which is not the recommended regimen for the treatment of severe malaria, was used due to the lack of parenteral antimalarial medications and the impossibility of providing them within a short period of time. Empirical therapy with IV ceftriaxone was also initiated, along with symptomatic, rehydration (2000 mL/day), and supportive therapy.

The patient was febrile for the first three days of hospitalization while being afebrile until the end of the treatment. The results of blood tests deteriorated during the first week of hospitalization and gradually improved during the second week. Because of the serious deterioration of renal functions, the patient underwent five cycles of dialysis starting from day 3 of hospitalization. Urine production improved from the 8th day of hospitalization and was normalized on discharge. 

After 14 days of intensive care, the patient was discharged in an improved health condition and normalized lab results, with red blood cell (RBC) count with a value of 3.95 × 10^12^/L (3.80–5.80 × 10^12^/L), hemoglobin (Hgb) level of 11.1 g/dL (11.00–16.5 g/dL), white blood cells (WBC) within the reference range with a count of 7.6 × 10^9^/L (3.5–10.0 × 10^9^/L), C-reactive protein (CRP) was 16.8 mg/dL, aspartate aminotransferase (AST) = 35 UI/L, alanine transaminase (ALT) = 40 UI/L, bilirubin total 10.2 µmol/L, direct bilirubin 4.3 µmol/L, urea 12.9 mmol/L, urea 12.9 mml/L, and creatinine 112 µmol/L.

Thin blood smear examination on the last day of hospitalization showed no signs of parasitemia. The physical examination and blood tests conducted two weeks after hospitalization show the patient’s complete clinical and laboratory recovery.

## 3. Discussion

The presented case highlights several important aspects of the prophylaxis failure as well as the diagnosis and management of malaria in non-endemic resource-limited countries. Despite receiving prophylactic doxycycline treatment, the patient experienced symptoms consistent with *Plasmodium falciparum* infection. Doxycycline is a commonly prescribed prophylactic medication for travelers visiting malaria-endemic regions. However, failures of doxycycline prophylaxis have been reported in the literature, suggesting a need for further evaluation of its efficacy [9,10]. In the presented case, the reasons for prophylactic and clinical failure of doxycycline against *P. falciparum* as inadequate dose and poor patient compliance due to simply forgetting and possible side effects, were excluded after a detailed history was taken. The delay in symptom onset after completing doxycycline prophylaxis has been described and should raise concerns about the potential for the delayed presentation of malaria [9]. The clinical presentation of our case is similar to cases of severe malaria with AKI presented in various studies and case presentations [19,20,21,22,23,24]. The laboratory findings in this case revealed significant abnormalities, including a low red blood cell count, elevated liver enzymes, and impaired renal function. These findings, together with a high level of parasitemia >10%, are consistent with the WHO criteria of severe malaria and the involvement of multiple organs in severe malaria cases [2,18,19,20,21,22,23,24]. The presence of *Plasmodium falciparum* growing rings and trophozoites on thin blood smears confirmed the diagnosis of falciparum malaria, known for its potentially severe and complicated course [2]. Prompt initiation of appropriate antimalarial therapy is crucial in severe malaria cases. In the case presented, the six-day course of oral artemether-lumefantrine resulted in clinical improvement and the resolution of symptoms, although this regimen is not recommended for the treatment of severe malaria [2]. The reason for using the regimen not recommended by the WHO for severe malaria was the lack of parenteral artesunate and artemether in Kosovo and the impossibility of obtaining medications from abroad within a reasonable time. This obstacle is well recognized by the World Health Organization (WHO) [2]. Supportive care played a vital role in managing this case. The patient also received antibiotics, IV fluids, several blood transfusions and transfusions of fresh frozen plasma. Five dialysis sessions were applied to the patient, which indicates the serious involvement of the kidneys, a recognized complication of severe malaria [18,19,20]. The importance of supportive treatment and dialysis in the treatment of cases of severe malaria with AKI has been proven once again in our case, described widely in different papers dealing with severe malaria with AKI [19,20,21,22,23,24,25,26,27]. The improvement of the patient’s condition is closely related to the immediate administration of antimalarial therapy (even oral) and the timely initiation of dialysis. 

Supportive therapy in our case is in line with WHO recommendations as well as experiences presented in various studies [2,17,18,21,22,23,24,25,26]. The study provides valuable insights into the clinical features and management of severe malaria cases, emphasizing the importance of intensive care unit management [14,20]. This case report also raises concerns about the efficacy of doxycycline prophylaxis. Similar reports of prophylaxis failure have been documented in the literature [9,10]. These findings call for further research and evaluation of alternative preventive strategies, especially in areas with drug-resistant strains. 

This case report is subject to some limitations. Firstly, we were not able to obtain the level of doxycycline concentration in plasma, due to the late hospitalization of the patient and lack of experience from the hospital staff, which would confirm the failure of prophylactic treatment. Secondly, there is a notable constraint in providing appropriate parenteral therapy for cases of severe malaria, primarily stemming from financial constraints and suboptimal operation of healthcare institutions in developing nations undergoing prolonged transitions, exemplified by the situation in Kosovo.

## 4. Conclusions

This case report highlights the challenges in diagnosing and treating severe malaria in a patient who had recently traveled to a malaria-endemic region. Despite receiving doxycycline prophylaxis, the patient developed symptoms of falciparum malaria. Healthcare providers should be aware of the potential for prophylaxis failure and consider the possibility of malaria even in patients who have regularly received prophylactic treatment. The presentation of this case also highlights the difficulties in diagnosing and treating malaria, especially severe malaria, in non-endemic countries with a lack of skilled manpower, quality microscopy, and parenteral antimalarial medications recommended by the WHO. In the presented case, the imposed treatment with oral artemether-lumefantrine resulted in the clinical improvement of the patient. 

## Figures and Tables

**Table 1:** Blood test results during the hospitalization.

Hospitalization/Day	RBC (×10^12^/L)	WBC (×10^9^/L)	Hgb (g/dL)	Hct (%)	Urea (mmol/L)	Creatinine (µmol/L)	CRP (mg/L)	AST (U/L)	ALT (U/L)
1	3.93	9.2	12.2	35	19.45	223.7	113.1	90	82
2	4.15	8.8	12.5	36.9	26.86	358.3	N/A	183	165
3	3.04	11.4	9.1	26.3	31.78	457.9	212.1	106	119
4	3.31	12.3	9.6	28.5	35.31	557.8	N/A	131	103
5	2.77	17.4	8.1	24.1	39.15	524.6	N/A	57	153
6	2.36	17.5	6.9	20.5	38.21	542.9	N/A	39	116
7	2.40	16.0	6.5	18	37.90	426.3	185.0	26	95
8	2.61	12.0	7.6	22.2	79.40	439.9	120.5	107	37
9	3.12	12.0	9	26.5	34.40	302.8	N/A	N/A	N/A
10	3.35	9.6	9.6	28.8	25.76	175.9	39.3	56	55
11	N/A	N/A	N/A	N/A	19.02	150.3	N/A	N/A	N/A
12	3.54	8.7	10.6	29.9	13.32	113.6	18.5	48	50
13	N/A	N/A	N/A	N/A	13.10	112.1	N/A	N/A	N/A
14	3.95	7.6	11.1	31.4	12.90	112	16.8	35	40

Abbreviations: RBC (Red Blood Cells), WBC (White Blood Cells), Hgb (Hemoglobin), Hct (Hematocrit), CRP (C—reactive protein), AST (Aspartate Aminotransferase), ALT (Alanine Transaminase), N/A (Not Available).

## Data Availability

Data presented in this case report are available on request from the corresponding author.
